# Development of Small Bowel Volvulus on Barbed V-Loc™ Suture: A Rare Complication after Laparoscopic Ventral Rectopexy

**DOI:** 10.1155/2018/8406054

**Published:** 2018-04-24

**Authors:** Giovanni Terrosu, Vittorio Cherchi, Umberto Baccarani, Gian Luigi Adani, Dario Lorenzin, Riccardo Pravisani, Serena Bertozzi, Sergio Calandra, Luigino Noce, Antonia Lavinia Zuliani, Andrea Risaliti

**Affiliations:** Department of Medicine, University of Udine, P.le Kolbe, 33100 Udine, Italy

## Abstract

In this case report, we share our experience with an emerging complication in laparoscopic surgery caused by the use of barbed sutures for an off-label indication. We describe a postoperative volvulus caused by the adhesion of the small bowel and V-Loc suture after a ventral laparoscopic rectopexy in a 48-year-old female patient. We also suggest cutting flush the end of the V-Loc and extending the follow-up of these patients.

## 1. Introduction

Laparoscopic techniques are becoming increasingly easier to perform, thanks to new materials, new approaches, and new tools that allow surgeons to be more time-effective and precise. In this case report, we want to focus on the barbed suture, a relatively new device that has been developed as a superficial suture but then has been proposed to facilitate laparoscopic procedures as a useful time-saving device.

This new technique involves the use of the standard suture material with axially spaced barbed segments on each side of a midpoint. At the midpoint, the barbs change direction allowing self-anchoring and a more uniform distribution of the tension, without the need to tie any knot [1].

However, the surgical world knows little about the adverse events associated with this new device, and nowadays, concerns have arisen regarding the safety of the barbed suture.

## 2. Case Report

A 48-year-old female patient, with BMI 20.3, presented to our hospital for a chronic constipation. The woman could only evacuate once a week despite laxatives. The defecation needed prolonged strain and anal digitation. She also complained about symptoms connected to a fractionated defecation. Her anamnesis showed a long history of depression, under treatment, and a solitary rectal ulcer.

A clinical diagnosis of obstructive defecation syndrome (Wexner constipation score = 14) and anterior anal fissure was made; the patient was then subject to colonoscopy and defecography that showed an anterior rectocele with an ampullary invagination.

A ventral laparoscopic rectopexy (d'Hoore technique) was performed. First, a right paraumbilical incision was made to introduce a Hasson trocar, which showed citric parauterine effusion and dolicosigma, and then, two other trocars were placed, one in the right lumbar region (10 mm) and the second in the left iliac region (5 mm).

The mesh (2 × 10 cm) was anchored inferiorly to the anterior wall of the rectum with propylene sutures and proximally to the sacral promontory with ProTack™. The peritoneal synthesis was made with a barbed suture (V-Loc) with a cut end of 1 cm.

The patient was discharged on day 3 after surgery, in good clinical conditions with a regular diet, regular bowel function, stable parameters, and without fever.

On day 8 after surgery, the patient was reevaluated: she showed no clinical issues and a proper healing of the scars.

On day 22 after surgery, the patient complained about strong abdominal pain and was subsequently studied with CT, which showed an abdominal occlusion due to a volvulus. The patient underwent surgery again: we performed explorative laparoscopy, revealing a high-grade obstruction from a volvulized loop of the distal ileum with distention, ischemic sufferance, and purulent effusion.

**Figure 1:**
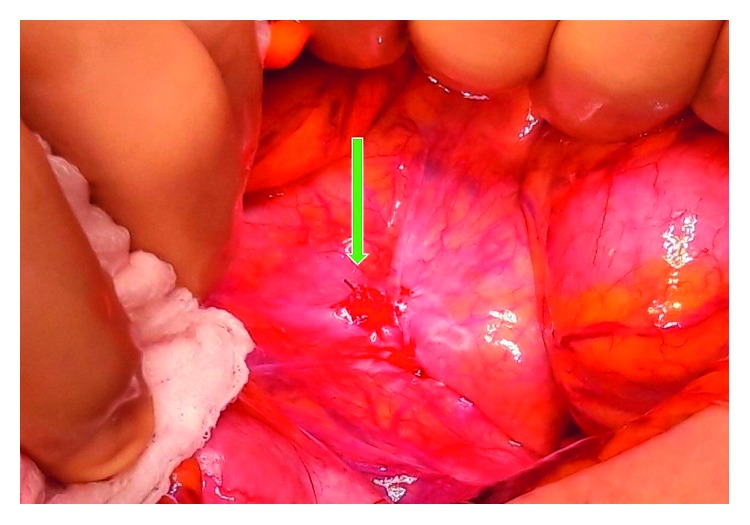
Adhesion between the peritoneum and the distal ileum in the Douglas' pouch.

**Figure 2:**
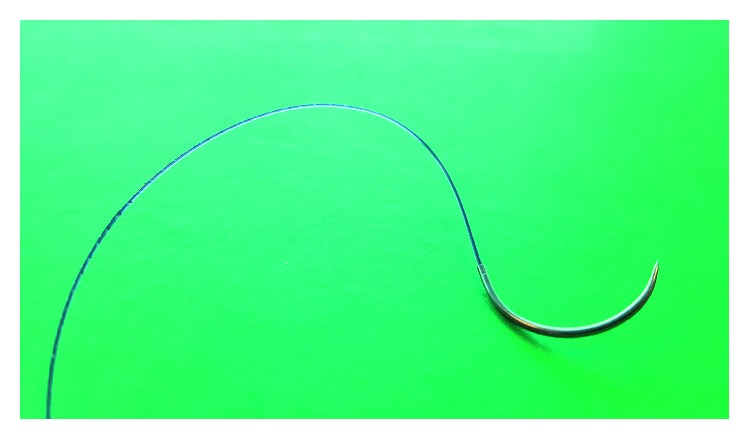
V-Loc barbed suture.

The procedure was converted to a laparotomy. The purulent material was sent for microbiological culture (negative for aerobic and anaerobic bacteria and yeast). An adhesion was found between the peritoneum and the distal ileum in the Douglas' pouch. It was caused by a flogistic and fibrotic reaction to the cut end of the barbed suture. A volvulus reduction was then performed, leading to a prompt reperfusion of the tract. The V-Loc was removed after the procedure, and the peritoneum was closed with introflecting Vicryl sutures. The patient was discharged after six days in good condition (Figures 1 and 2).

## 3. Discussion

The suture we are describing in this report is designed to pass through the tissues and keep them together with the needed tension, avoiding loosening. This is possible, thanks to the barbed segments that are at the same time the reason why the observed condition takes place: the volvulus develops where the viscera touches the barbed suture, and there, we find the axis of the twisted organ. When we came across this patient's condition, we were unaware of the specific connection between the barbed suture and intestinal volvulus, so we decided to perform a research on PubMed, looking for similar cases. We found case reports mainly referring to gynecological surgery [2–5], but we also found some reports about rectopexy [6, 7]. It was very interesting to compare the different timing before the manifestation of this complication: some patients developed small bowel obstruction in less than a week and some others after one to four months.

Segura-Sampedro et al. [8] collected 15 cases of small bowel obstruction due to laparoscopic use of barbed sutures in literature. It is interesting to highlight that all the cases analyzed occurred in patients undergoing surgery below the transverse colon, suggesting that further studies are required to be sure that the use of the barbed suture in inframesocolic surgery is actually not a contraindication.

Segura-Sampedro and other surgeons [6–8] who came across this complication suggested that a possible solution may be reducing the cut end of the V-Loc or covering the cut end with a cellulose sheath or a laparoscopic clip. While this seems a reasonable approach, there is a study conducted by Sakata et al. [9] which shows the possibility of an exposure of barbs in the pelvic cavity even if the suture is cut flush due to the fibrotic reaction of the peritoneum. Sakata et al. conclude that the risk is unavoidable and suggests a return to manual sutures.

## 4. Conclusion

It is undeniable that barbed sutures are improving surgical efficiency, but the possibility for the barbs to attach to surrounding tissue is a cause for actual concern. It is important to try to avoid this complication by performing a cut flush end, even though it may still not be sufficient to remove the risk completely. Consequently, it is also of paramount importance to follow up patients who have undergone this kind of procedure, knowing that complications may arise in a large span of time.

In our clinical practice, we think that this particular suture should be used cautiously, covering every segment of it and performing at the end of the procedure a cut flush end. No segments of the suture should be able to interact with the intestines.

Finally, we think that this condition may require a systematic study, in order to collect all data and find out the actual incidence of volvulus on the barbed suture, pointing out possible specific contraindications to this technique.

## Data Availability

Our data are stored in the hospital system and available via e-mail if required.
